# Supplementary material for: Using LSTM and PSO techniques for predicting moisture content of poplar fibers by Impulse-cyclone Drying
Source: PLoS One. 2022 Apr 11;17(4):e0266186. doi: 10.1371/journal.pone.0266186 (PMC9000088; doi:10.1371/journal.pone.0266186)
Supplement: S1 File — (DOCX) [file pone.0266186.s001.docx]

**Supporting Information**

Using LSTM and PSO Techniques for Predicting Moisture Content of Poplar Fibers by Impulse-cyclone Drying

Feng Chen^1^*, Xun Gao^2&^, Xinghua Xia^1^, Jing Xu^3&^

1. School of Art and Design, Taizhou University, Taizhou, Zhejiang, China
2. College of Civil Engineering, Hunan University, Changsha, Hunan, China
3. College of Material Science and Engineering, Northeast Forestry University, Harbin, Heilongjiang, China

* Corresponding author

E-mail: [chenfeng1984@tzc.edu.cn](mailto:chenfeng1984@tzc.edu.cn)

^&^ These authors contributed equally to this work.

**Figure S1. Impulse-cyclone drying system used in the study.**

At the beginning of the drying test, warm air was injected into the (1) electric heater to preheat the drying system. After the system was filled with hot air and inlet temperature was constant, the (9) screw feeder was used to begin pressing the wood fibers into the (2) impulse dryer. Under the accelerating and decelerating motion of wood fibers in the impulse dryer, free water in wood cell cavity is quickly vaporized within the impulse dryer. The fibers were allowed to flow into the (3) cyclone dryer to remove the water bound to the surface of the pulp, which was difficult to dry. The larger fibers were collected by the (8) air-off layout collector and the finer particles entered the (5) dust collector with the exhaust gas. In order to verify the temperature change and airflow velocity change in ICAD system during the simulated drying process, temperature measuring thermocouples and velocity sensors were placed at the inlet, straight tube, impulse tube, middle and gas outlet of the drying system, (shown by A, B, C, D and E in Figure S1).


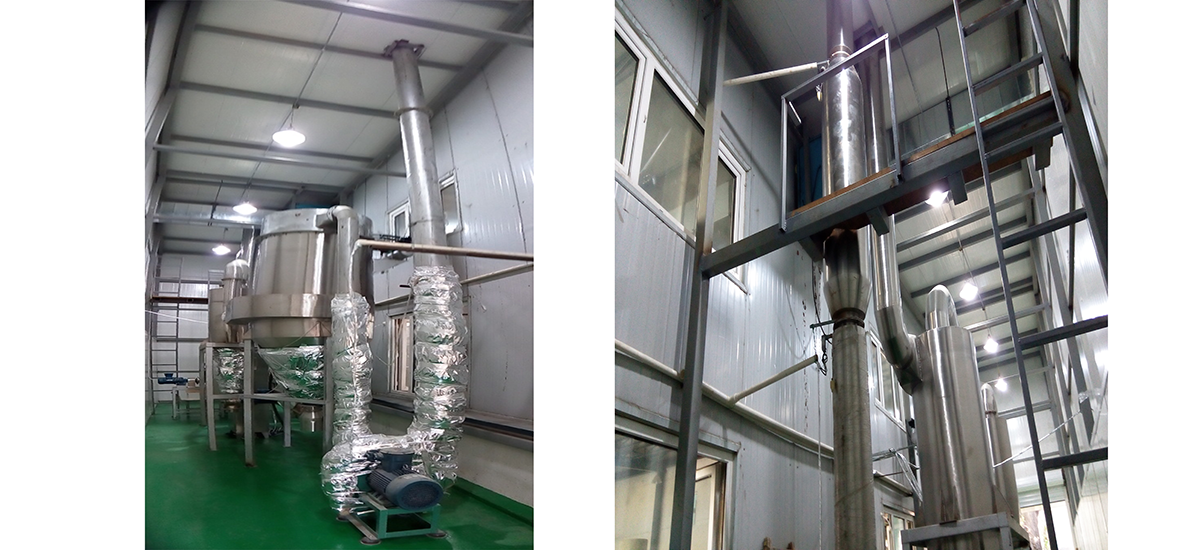


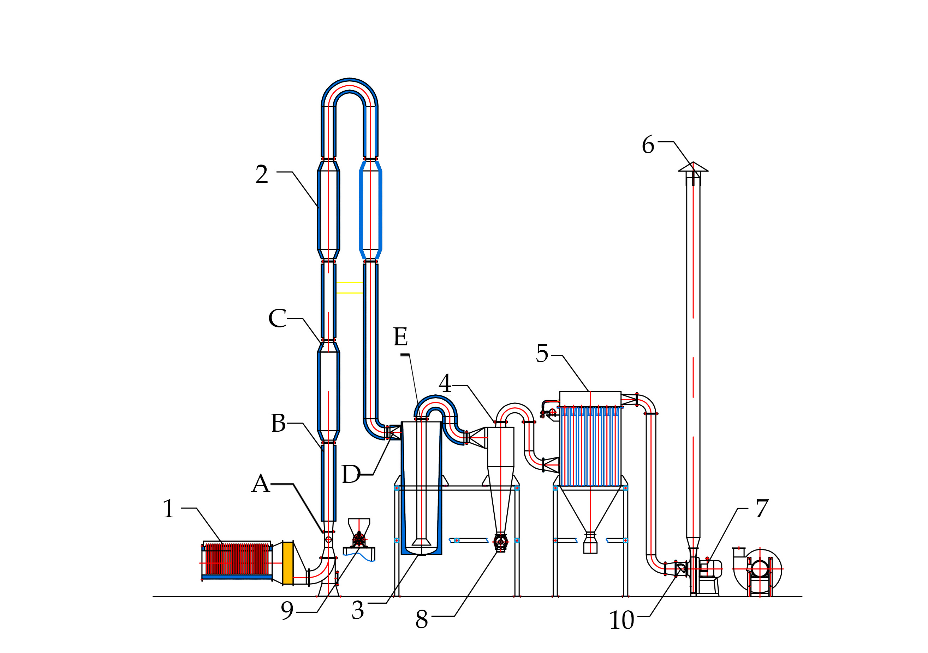


**Figure S1. Functional diagram of impulse- cyclone airflow drying device: (1) Electric heater; (2) Impulse drier; (3) Cyclone drier; (4) Cyclone separator; (5) Dust collector; (6) Exhaust pipe; (7) Induced draft fan; (8) Air-off layout collector; (9) Screw feeder; and (10) Air valve.**

References

- Feng C., Li Q., Xun G., Han G., Cheng W. Impulse-cyclone Drying Treatment of Poplar Wood Fibers and its Effect on Composite Material's Properties. Bioresources. 2017;12(2): 3948-3964. DOI: 10.15376/biores.12.2.3948-3964

**Figure S2. Biomass fiber crusher.**

Because poplar veneers are easier to obtain in a disintegrator with uniform aspect ratio and MC after milling, poplar veneers of about 500 kg (Zhonghan-17 fast-growing poplar, Harbin Yongxu Wood-Based Panel Co., Ltd., Heilongjiang, China) were selected as the fiber source. The size of each veneer was 1.2 mm × 40 mm × 40 mm, the air-dried density was 0.38 g/m^3^, and the average MC was 13.8 (± 1.2) %. To satisfy the experimental requirements, the veneers were crushed into 60–80 mesh fiber samples by a biomass fiber crusher (MF-600, Jiangsu Fuyang Machinery Co., Ltd., Xuzhou, China).


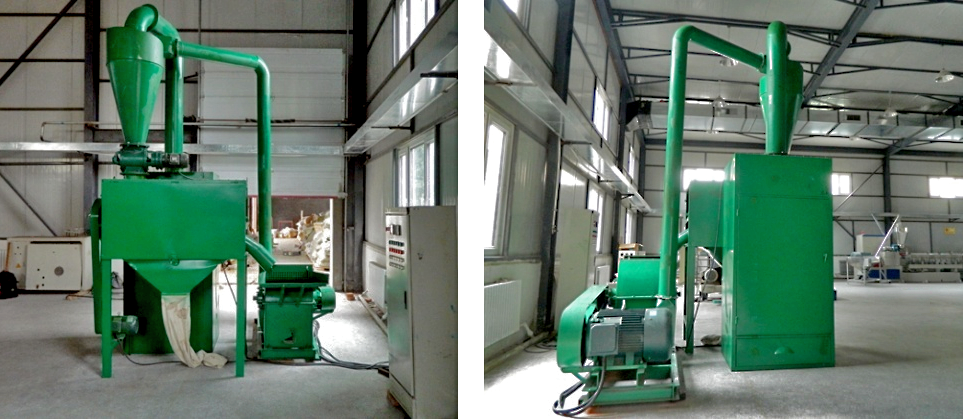


**Figure S2. Biomass fiber crusher.**

References

- Xun G., Li Q., Cheng W., Han G., Xuan L. Optimization of High Temperature and Pressurized Steam Modified Wood Fibers for High-Density Polyethylene Matrix Composites Using the Orthogonal Design Method. Materials. 2016; 9(10): 847. DOI: 10.3390/ma9100847.

**Table S1. The measurement process of wood fiber morphology.**

The length and diameter of fibers were measured by a GE-5 high-definition digital microscope (Shanghai Longfa Optical Instrument Co., Ltd.). The different meshes of wood fibers were placed under the objective lens of the microscope, which was adjusted to present a well-focus image. The wood fibers on the slide were measured for their length and diameter values through the measurement software of the microscope. Thirty pieces of data were measured for each mesh segment, and the average aspect ratio of the wood fibers was then determined. The measured results of fiber length, fiber diameter and original moisture content are given in Table S4-1.

**Table S1.** Characteristics of Poplar Wood Fibers

| **Property Measured (meshes)** | **Fiber Length (mm)** | **Fiber Diameter (μm)** | **Length-diam. Ratio** | **MC at 23 °C (%)** |
| --- | --- | --- | --- | --- |
| *20- to 40-* | 1.78 (±0.23) | 330 (±51) | 5.4 (±1.95) | 13.8 (±1.2) |
| *40- to 60-* | 1.53 (±0.18) | 283 (±35) | 5.4 (±1.61) |  |
| *60- to 80-* | 1.36 (±0.20) | 223 (±31) | 6.1 (±1.87) |  |
| *80- to 100-* | 0.94 (±0.16) | 192 (±47) | 4.9 (±1.44) |  |
| *100- to 120-* | 0.64 (±0.13) | 178 (±33) | 3.6 (±0.97) |  |

**Table S2. RSM experimental design with the independent parameters.**

The results of the RSM experiment were analyzed by Design Expert 10.0.4, and the data of the RSM experiment were fitted with the multiple regression model analyzed via analysis of variance (ANOVA). The results are shown in Table S2

**Table S2. The results of RSM experimental**

| **S.no.** | **A-Initial MC (%)** | **B-Inlet air temperature (°C)** | **C-Feed rate (kg/h)** | **D-Inlet air velocity (m/s)** | **Y-Final MC (%)** |
| --- | --- | --- | --- | --- | --- |
| *1.* | 240 | 11 | 120 | 20 | 2.81 |
| *2.* | 180 | 12 | 135 | 10 | 3.2 |
| *3.* | 200 | 11 | 150 | 20 | 4.5 |
| *4.* | 220 | 10 | 105 | 10 | 2.1 |
| *5.* | 180 | 10 | 135 | 10 | 2.8 |
| *6.* | 200 | 13 | 120 | 20 | 5.4 |
| *7.* | 200 | 11 | 120 | 20 | 4.1 |
| *8.* | 200 | 11 | 120 | 20 | 4 |
| *9.* | 220 | 10 | 135 | 30 | 6.8 |
| *10.* | 160 | 11 | 120 | 20 | 7.4 |
| *11.* | 180 | 10 | 105 | 10 | 1.8 |
| *12.* | 200 | 11 | 120 | 20 | 5.6 |
| *13.* | 180 | 12 | 105 | 10 | 2.6 |
| *14.* | 180 | 12 | 135 | 30 | 9.4 |
| *15.* | 200 | 11 | 120 | 20 | 3.9 |
| *16.* | 180 | 10 | 135 | 30 | 7.8 |
| *17.* | 220 | 12 | 135 | 30 | 4.5 |
| *18.* | 200 | 11 | 120 | 0 | 0 |
| *19.* | 200 | 11 | 120 | 20 | 3.9 |
| *20.* | 180 | 10 | 105 | 30 | 6.4 |
| *21.* | 220 | 10 | 105 | 30 | 5.1 |
| *22.* | 220 | 10 | 135 | 10 | 2.8 |
| *23.* | 200 | 11 | 120 | 20 | 4.2 |
| *24.* | 220 | 12 | 105 | 10 | 1.1 |
| *25.* | 220 | 12 | 135 | 10 | 1.8 |
| *26.* | 180 | 12 | 105 | 30 | 7.2 |
| *27.* | 200 | 11 | 90 | 20 | 3.2 |
| *28.* | 200 | 11 | 120 | 40 | 9.5 |
| *29.* | 200 | 9 | 120 | 20 | 2 |
| *30.* | 220 | 12 | 105 | 30 | 3.4 |

**Table S3. Variance analysis of regression model.**

The results of the RSM experiment were analyzed by Design Expert 10.0.4, and the data of the RSM experiment were fitted with the multiple regression model analyzed by ANOVA. The results of ANOVA are shown in Table S3. The *F*-value of the model is 71.86, and the *P*-value is lower than 0.0001, indicating that the model is significant.

**Table S3. Variance analysis of regression model.**

| **Source** | **Sum of Squares** | **Degree of freedom** | **Mean Square** | ***F* value** | ***P*-value** |  |
| --- | --- | --- | --- | --- | --- | --- |
| *Model* | 88.15 | 14 | 6.30 | 71.86 | ＜0.0001 | Significant |
| *A* | 110.08 | 1 | 110.08 | 164.06 | ＜0.0001 |  |
| *B* | 21.62 | 1 | 21.62 | 32.22 | ＜0.0001 |  |
| *C* | 6.00 | 1 | 6.00 | 8.94 | 0.0075 |  |
| *D* | 0.81 | 1 | 0.81 | 1.20 | 0.2866 |  |
| *AB* | 4.41 | 1 | 4.41 | 6.57 | 0.0190 |  |
| *AC* | 0.72 | 1 | 0.72 | 1.08 | 0.3125 |  |
| *AD* | 0.040 | 1 | 0.040 | 0.060 | 0.8097 |  |
| *BC* | 0.063 | 1 | 0.063 | 0.093 | 0.7635 |  |
| *CD* | 2.500E-003 | 1 | 2.500E-003 | 3.726E-003 | 0.9520 |  |
| *A2* | 0.90 | 1 | 0.90 | 20.63 | 0.0003 |  |
| *B2* | 0.072 | 1 | 0.072 | 0.78 | 0.3784 |  |
| *C2* | 0.025 | 1 | 0.025 | 0.72 | 0.4253 |  |
| *D2* | 1.27 | 1 | 1.27 | 13.38 | 0.0014 |  |
| *Residual* | 12.75 | 19 | 0.67 |  |  |  |
| *Lack of Fit* | 10.60 | 14 | 0.76 | 1.76 | 0.2762 | Not Significant |
| *Pure Error* | 2.15 | 5 | 0.43 |  |  |  |
| *Cor Total* | 162.26 | 29 |  |  |  |  |

**Table S4. Single-Factor Results.**

Table S4-1 showed the change concerning the final moisture content of poplar fiber with the inlet air temperature. Table S4-2showed the variation in the final moisture content of poplar fiber with the air inlet velocity. Table S4-3showed the change concerning the final moisture content of poplar fiber with the feed rate.

**Table S4-1.** **Effects of different inlet air temperatures on final moisture content.**

| **Temperature (^o^C)** | **Initial MC (%)** | **Final MC (%)** | **Initial MC (%)** | **Final MC (%)** | **Initial MC (%)** | **Final MC (%)** |
| --- | --- | --- | --- | --- | --- | --- |
| *120* | 12.4 | 4.3±0.2 | 31.8 | 7.7±0.4 | 51.6 | 10.9±0.4 |
| *140* | 12.4 | 3.6±0.3 | 31.8 | 6.3±0.2 | 51.6 | 8.9±0.4 |
| *160* | 12.4 | 2.9±0.4 | 31.8 | 5.3±0.4 | 51.6 | 8.2±0.3 |
| *180* | 12.4 | 2.4±0.2 | 31.8 | 4.8±0.2 | 51.6 | 7.7±0.2 |
| *200* | 12.4 | 1.9±0.2 | 31.8 | 4.3±0.2 | 51.6 | 7.1±0.3 |
| *220* | 12.4 | 1.4±0.1 | 31.8 | 3.9±0.3 | 51.6 | 6.5±0.2 |
| *240* | 12.4 | 1.1±0.1 | 31.8 | 3.5±0.2 | 51.6 | 6.1±0.4 |

**Table S4-2.** **Effects of different inlet air velocities on final moisture content.**

| **Inlet airflow velocity (m/s)** | **Initial MC (%)** | **Final MC (%)** | **Initial MC (%)** | **Final MC (%)** | **Initial MC (%)** | **Final MC (%)** |
| --- | --- | --- | --- | --- | --- | --- |
| *7* | 12.4 | 1.0±0.1 | 31.8 | 1.0±0.1 | 51.6 | 1.2±0.1 |
| *8* | 12.4 | 1.1±0.1 | 31.8 | 1.1±0.1 | 51.6 | 1.3±0.1 |
| *9* | 12.4 | 1.4±0.2 | 31.8 | 2.1±0.2 | 51.6 | 3.1±0.4 |
| *10* | 12.4 | 1.8±0.4 | 31.8 | 3.2±0.2 | 51.6 | 5.2±0.2 |
| *11* | 12.4 | 1.9±0.3 | 31.8 | 4.3±0.2 | 51.6 | 7.1±0.2 |
| *12* | 12.4 | 2.3±0.4 | 31.8 | 6.6±0.2 | 51.6 | 9.8±0.4 |
| *13* | 12.4 | 2.4±0.4 | 31.8 | 9.5±0.4 | 51.6 | 14.1±0.3 |

**Table S4-3.** **Effects of different feed rates on final moisture content.**

| **Feed rate (kg/h)** | **Initial MC (%)** | **Final MC (%)** | **Initial MC (%)** | **Final MC (%)** | **Initial MC (%)** | **Final MC (%)** |
| --- | --- | --- | --- | --- | --- | --- |
| *60* | 12.4 | 0.6±0.2 | 31.8 | 2.1±0.1 | 51.6 | 2.6±0.2 |
| *75* | 12.4 | 0.8±0.3 | 31.8 | 2.5±0.2 | 51.6 | 3.1±0.2 |
| *90* | 12.4 | 1.0±0.1 | 31.8 | 3.0±0.1 | 51.6 | 4.1±0.3 |
| *105* | 12.4 | 1.5±0.4 | 31.8 | 3.6±0.3 | 51.6 | 5.4±0.2 |
| *120* | 12.4 | 1.9±0.1 | 31.8 | 4.3±0.4 | 51.6 | 7.1±0.2 |
| *135* | 12.4 | 2.4±0.2 | 31.8 | 5.5±0.2 | 51.6 | 9.2±0.4 |
| *150* | 12.4 | 2.5±0.2 | 31.8 | 6.1±0.4 | 51.6 | 10.5±0.1 |

**Table S5. Comparative analysis on experimental predicted values of three models.**

| **S.no.** | **RSM** | **BP** | **LSTM** | **Actual MC** |
| --- | --- | --- | --- | --- |
| *1* | 1.23 | 0.67 | 0.96 | 1.0 |
| *2* | 1.41 | 1.75 | 2.42 | 2.0 |
| *3* | 2.55 | 3.31 | 2.51 | 2.5 |
| *4* | 4.03 | 4.45 | 4.42 | 4.4 |
| *5* | 3.91 | 4.57 | 4.61 | 5.2 |
| *6* | 5.58 | 5.78 | 4.75 | 5.4 |
| *7* | 3.82 | 4.72 | 4.81 | 6.2 |
| *8* | 3.75 | 4.81 | 5.06 | 6.3 |
| *9* | 7.84 | 6.21 | 6.37 | 7.0 |
| *10* | 9.04 | 7.77 | 7.91 | 7.7 |
| *11* | 7.84 | 9.26 | 8.12 | 8.9 |
| *12* | 8.54 | 8.62 | 8.71 | 9.5 |
| *R^2^* | 0.8801 | 0.9363 | 0.9446 |  |
| *r* | 0.8715 | 0.935 | 0.9809 |  |
| *MSE* | 0.3647 | 0.1665 | 0.2316 |  |
| *MAE* | 0.3911 | 0.302 | 0.2207 |  |
| *MAPE* | 0.6876 | 0.437 | 0.4124 |  |

**Table S6. Comparison and Verification of Different Methods with 96 groups test data for predicting MC.**

To verify the accuracy of the proposed scheme, LSTM, and PSO-LSTM models were used to predict the MC on the same data set. By comparing 96 groups of data for the test set, it can be found that the results simulated by PSO-LSTM were better than those of LSTM model (Table S6).

**Table S6. Comparison and Verification of Different Methods with 96 groups test data for predicting MC.**

| **S.no.** | **LSTM** | **POS-LSTM** | **Actual MC** |
| --- | --- | --- | --- |
| *1* | 0.89 | 1.09 | 0.69 |
| *2* | 1.13 | 1.41 | 0.98 |
| *3* | 1.13 | 1.54 | 1.40 |
| *4* | 1.15 | 1.57 | 1.97 |
| *5* | 1.26 | 1.56 | 1.27 |
| *6* | 1.26 | 1.54 | 1.20 |
| *7* | 1.27 | 1.58 | 1.58 |
| *8* | 1.28 | 1.58 | 1.59 |
| *9* | 1.37 | 1.69 | 1.36 |
| *10* | 1.46 | 1.75 | 1.67 |
| *11* | 1.74 | 2.06 | 1.86 |
| *12* | 1.78 | 2.06 | 1.68 |
| *13* | 1.83 | 2.16 | 1.78 |
| *14* | 1.93 | 2.24 | 2.65 |
| *15* | 2.20 | 2.27 | 1.95 |
| *16* | 2.09 | 2.33 | 2.13 |
| *17* | 2.14 | 2.44 | 2.33 |
| *18* | 2.35 | 2.63 | 2.33 |
| *19* | 2.58 | 2.85 | 2.81 |
| *20* | 2.64 | 2.97 | 3.25 |
| *21* | 2.76 | 3.05 | 3.23 |
| *22* | 2.81 | 3.18 | 3.21 |
| *23* | 8.15 | 7.87 | 6.97 |
| *24* | 8.16 | 7.84 | 7.34 |
| *25* | 8.27 | 7.92 | 7.89 |
| *26* | 8.25 | 7.94 | 7.27 |
| *27* | 8.39 | 8.08 | 8.07 |
| *28* | 8.51 | 8.26 | 8.07 |
| *29* | 8.62 | 8.32 | 8.24 |
| *30* | 8.72 | 8.43 | 8.60 |
| *31* | 8.84 | 8.54 | 8.05 |
| *32* | 8.85 | 8.55 | 7.92 |
| *33* | 9.08 | 8.75 | 8.59 |
| *34* | 9.19 | 8.89 | 8.59 |
| *35* | 9.23 | 8.93 | 9.03 |
| *36* | 9.35 | 9.04 | 9.01 |
| *37* | 9.34 | 9.08 | 9.46 |
| *38* | 9.43 | 9.18 | 9.16 |
| *39* | 9.58 | 9.22 | 10.83 |
| *40* | 10.21 | 9.53 | 8.7 |
| *41* | 10.52 | 9.85 | 9.75 |
| *42* | 10.96 | 11.89 | 11.11 |
| *43* | 11.51 | 11.47 | 12.20 |
| *44* | 11.86 | 11.33 | 11.92 |
| *45* | 11.98 | 11.25 | 11.72 |
| *46* | 11.95 | 11.14 | 10.88 |
| *47* | 12.12 | 11.13 | 11.20 |
| *48* | 12.89 | 10.86 | 10.13 |
| *49* | 13.11 | 10.81 | 10.45 |
| *50* | 13.19 | 10.81 | 10.75 |
| *51* | 13.27 | 10.75 | 12.65 |
| *52* | 13.24 | 10.73 | 10.91 |
| *53* | 13.38 | 10.65 | 10.2 |
| *54* | 13.35 | 10.51 | 10.38 |
| *55* | 13.56 | 10.34 | 12.81 |
| *56* | 14.65 | 10.32 | 10.45 |
| *57* | 14.81 | 10.24 | 10.88 |
| *58* | 14.87 | 10.13 | 10.35 |
| *59* | 14.82 | 7.21 | 7.93 |
| *60* | 14.91 | 7.77 | 11.86 |
| *61* | 1.26 | 3.32 | 3.99 |
| *62* | 1.26 | 3.23 | 2.93 |
| *63* | 1.27 | 3.38 | 3.76 |
| *64* | 1.33 | 3.42 | 3.18 |
| *65* | 1.58 | 3.50 | 3.6 |
| *66* | 1.71 | 3.67 | 3.9 |
| *67* | 1.77 | 3.87 | 3.7 |
| *68* | 1.91 | 3.49 | 3.46 |
| *69* | 1.94 | 3.98 | 3.59 |
| *70* | 5.19 | 7.27 | 7.4 |
| *71* | 5.18 | 7.21 | 6.73 |
| *72* | 5.05 | 7.12 | 6.5 |
| *73* | 4.89 | 6.93 | 7.01 |
| *74* | 4.01 | 6.93 | 6.35 |
| *75* | 4.82 | 6.80 | 6.92 |
| *76* | 4.33 | 6.35 | 6.25 |
| *77* | 4.03 | 6.03 | 6.51 |
| *78* | 4.00 | 6.11 | 6.04 |
| *79* | 4.95 | 5.87 | 6.41 |
| *80* | 4.54 | 5.54 | 5.01 |
| *81* | 4.53 | 5.54 | 5.22 |
| *82* | 4.48 | 5.51 | 5.48 |
| *83* | 4.36 | 5.42 | 5.43 |
| *84* | 4.41 | 5.34 | 6.11 |
| *85* | 4.22 | 5.38 | 5.37 |
| *86* | 4.10 | 5.07 | 4.62 |
| *87* | 4.08 | 5.06 | 4.67 |
| *88* | 3.01 | 4.15 | 4.36 |
| *89* | 3.15 | 4.21 | 3.86 |
| *90* | 3.44 | 4.40 | 3.95 |
| *91* | 3.41 | 4.18 | 4.42 |
| *92* | 3.51 | 4.48 | 4.53 |
| *93* | 3.66 | 4.68 | 4.79 |
| *94* | 3.84 | 4.82 | 4.48 |
| *95* | 2.88 | 4.91 | 4.4 |
| *96* | 2.99 | 5.12 | 4.88 |

**Table S7. BP, LSTM, PSO neural network described in Python language**

Based on Python language, the TensorFlow2.0 learning framework is used to study and construct the neural network model. The TensorFlow2.0 library was loaded into Anaconda in advance, and then the numpy, pandas and matplotlib libraries in Python data analysis were imported (Table S7).

**Table S7 BP, LSTM, PSO neural network described in Python language**

| **Model** | **Python Code** |
| --- | --- |
| *BP neural network* | import matplotlib.pyplot as plt  import pandas as pd  from numpy import array  from tensorflow.keras import layers, Sequential  import numpy as np  data = pd.read_csv("./1234.csv")  total = len(data)  print("sum of data:%d" % total)  des = data.describe()  Y = [ ]  X = [ ]  x1 = [ ]  for i in range(len(data.index)):  Y.append(data.e[i])  X_train = data.values  for i in range(len(X_train)):  for j in range(len(X_train[0]) - 1):  x1.append(X_train[i][j])  X.append(x1)  x1 = []  print(X)  n_steps = 4  n_features = 1  X = array(X)  Y = array(Y)  X_MAX,X_MIN = X.max(0) , X.min(0)  Y_MAX,Y_MIN = Y.max(0) , Y.min(0)  X = (X - X_MIN) / (X_MAX - X.min(0))# min max Normalization  Y = (Y - Y.min(0)) / (Y.max(0) - Y.min(0))  ds_x = array(X[:30])  ds_y = array(Y[:30])  test_x = array(X[30:])  test_y = array(Y[30:])  model = Sequential()  model.add(layers.Dense(50, activation='relu', input_shape=(n_steps * n_features,)))  model.add(layers.Dense(50, activation='relu'))  model.add(layers.Dense(1))  model.summary()  model.compile(optimizer='adam', loss='mse')  ds_x = ds_x.reshape((ds_x.shape[0], ds_x.shape[1]))  model.save("bpnet.h5")  history = model.fit(ds_x, ds_y, epochs=100)  loss = history.history['loss']  plt.figure(dpi=600)  # plt.figure(figsize = (10.3))  plt.plot(loss,color = 'red')  plt.ylim((-0.01,0.25))  plt.legend()  plt.show()  ds_y_pred = model.predict(ds_x)  ds_y_pred = ds_y_pred.flatten() * (Y_MAX - Y_MIN) + Y_MIN # fan gui yi hua  ds_y = ds_y * (Y_MAX - Y_MIN) + Y_MIN  plt.plot(ds_y_pred,label='train_y_pred', marker = 'o')  plt.plot(ds_y,label='train_y_true', marker = 'o')  plt.legend()  plt.show()  print(ds_y_pred)  print(ds_y)  mse1 = np.sum((ds_y - ds_y_pred) ** 2) / len(ds_y)  rmse1 = np.sqrt(mse1)  mae1 = np.sum(np.absolute(ds_y - ds_y_pred)) / len(ds_y)  r21 = 1-mse1/ np.var(ds_y)  print("mse:",mse1," rmse:",rmse1," mae:",mae1," r2:",r21)  print(np.corrcoef(ds_y, ds_y_pred))  y_pred = model.predict(test_x)  y_pred = y_pred.flatten() * (Y_MAX - Y_MIN) + Y_MIN # fan gui yi hua  test_y = test_y * (Y_MAX - Y_MIN) + Y_MIN  plt.plot(y_pred,label='test_y_pred', marker = 'x')  plt.plot(test_y,label='test_y_true', marker = 'x')  plt.legend()  plt.show()  y_pred.sort()  test_y.sort()  print(y_pred)  print(test_y)  mse = np.sum((test_y - y_pred) ** 2) / len(test_y)  rmse = np.sqrt(mse)  mae = np.sum(np.absolute(test_y - y_pred)) / len(test_y)  r2 = 1-mse/ np.var(test_y)  print("mse:",mse," rmse:",rmse," mae:",mae," r2:",r2)  print(np.corrcoef(test_y, y_pred)) |
| *LSTM neural network* | import matplotlib.pyplot as plt  import pandas as pd  from numpy import array  from tensorflow.keras import layers, Sequential  import numpy as np  data = pd.read_csv("./1234.csv")  total = len(data)  print("sum of data:%d" % total)  des = data.describe()  Y = []  X = []  x1 = []  for i in range(len(data.index)):  Y.append(data.e[i])  X_train = data.values  for i in range(len(X_train)):  for j in range(len(X_train[0]) - 1):  x1.append(X_train[i][j])  X.append(x1)  x1 = []  print(X)  n_steps = 4  n_features = 1  X = array(X)  Y = array(Y)  X_MAX,X_MIN = X.max(0) , X.min(0)  Y_MAX,Y_MIN = Y.max(0) , Y.min(0)  X = (X - X_MIN) / (X_MAX - X.min(0))# min max Normalization  Y = (Y - Y.min(0)) / (Y.max(0) - Y.min(0))  ds_x = np.expand_dims(X[:30],-1)  ds_y = Y[:30]  test_x = np.expand_dims(array(X[30:]),-1)  test_y = array(Y[30:])  model = Sequential()  model.add(layers.LSTM(50, activation='relu', input_shape=(n_steps, n_features)))  model.add(layers.Dense(1))  model.summary()  model.compile(optimizer='adam', loss='mse')  model.save("lstmnet.h5")  history = model.fit(ds_x, ds_y, epochs=5000)  loss = history.history['loss']  # plt.figure(figsize = (10.3))  plt.figure(dpi=600)  plt.plot(loss)  plt.ylim((-0.01,0.25))  plt.legend()  plt.show()  ds_y_pred = model.predict(ds_x)  ds_y_pred = ds_y_pred.flatten() * (Y_MAX - Y_MIN) + Y_MIN # fan gui yi hua  ds_y = ds_y * (Y_MAX - Y_MIN) + Y_MIN  plt.plot(ds_y_pred,label='train_y_pred', marker = 'o')  plt.plot(ds_y,label='train_y_true', marker = 'o')  plt.legend()  plt.show()  print(ds_y_pred)  print(ds_y)  mse1 = np.sum((ds_y - ds_y_pred) ** 2) / len(ds_y)  rmse1 = np.sqrt(mse1)  mae1 = np.sum(np.absolute(ds_y - ds_y_pred)) / len(ds_y)  r21 = 1-mse1/ np.var(ds_y)  print("mse:",mse1," rmse:",rmse1," mae:",mae1," r2:",r21)  print(np.corrcoef(ds_y, ds_y_pred))  y_pred = model.predict(test_x)  y_pred = y_pred.flatten() * (Y_MAX - Y_MIN) + Y_MIN # fan gui yi hua  test_y = test_y * (Y_MAX - Y_MIN) + Y_MIN  plt.plot(y_pred,label='test_y_pred', marker = 'x')  plt.plot(test_y,label='test_y_true', marker = 'x')  plt.legend()  plt.show()  y_pred.sort()  test_y.sort()  print(y_pred)  print(test_y)  mse = np.sum((test_y - y_pred) ** 2) / len(test_y)  rmse = np.sqrt(mse)  mae = np.sum(np.absolute(test_y - y_pred)) / len(test_y)  r2 = 1-mse/ np.var(test_y)  print("mse:",mse," rmse:",rmse," mae:",mae," r2:",r2)  print(np.corrcoef(test_y, y_pred)) |
| *PSO* | import tensorflow as tf  import numpy as np  import matplotlib.pyplot as plt  import pandas as pd  from sklearn.preprocessing import MinMaxScaler  import time  import random  INPUT_SIZE = 4  OUTPUT_SIZE = 1  # (2) PSO Parameters  MAX_EPISODES = 20  MAX_EP_STEPS = 100  c1 = 2  c2 = 2  w = 0.4  pN = 20  dim = 4  X = np.zeros((pN, dim))  V = np.zeros((pN, dim))  pbest = np.zeros((pN, dim))  gbest = np.zeros(dim)  p_fit = np.zeros(pN)  t1 = time.time()  # CELL_SIZE, LR ,TIME_STEP, BATCH_SIZE  UP = [40, 0.1, 30, 120]  DOWN = [5, 0.0001, 2, 10]  for i_episode in range(MAX_EPISODES):  random.seed(8)  fit = 0.2  print("Calculate the initial global optimum ")  for i in range(pN):  for j in range(dim):  V[i][j] = random.uniform(0, 1)  if j == 1:  X[i][j] = random.uniform(DOWN[j], UP[j])  else:  X[i][j] = round(random.randint(DOWN[j], UP[j]), 0)  pbest[i] = X[i]  le, pred = training(INPUT_SIZE, OUTPUT_SIZE, X[i])  NN = 1  tmp = function(pred, testing_target[:le], le)  p_fit[i] = tmp  if tmp > fit:  fit = tmp  gbest = X[i]  print("Calculate the initial global optimum：{:}".format(gbest))  fitness = []  for j in range(MAX_EP_STEPS):  fit2 = []  plt.title("Iteration".format(i_episode))  for i in range(pN):  le, pred = training(INPUT_SIZE, OUTPUT_SIZE, X[i])  temp = function(pred, testing_target[:le], le)  fit2.append(temp/1000)  if temp > p_fit[i]:  p_fit[i] = temp  pbest[i] = X[i]  if p_fit[i] > fit:  gbest = X[i]  fit = p_fit[i]  print("search steps：{:}".format(j))  print("Individual optimal parameters：{:}".format(pbest))  print("Global optimal parameters：{:}".format(gbest))  for i in range(pN):  V[i] = w * V[i] + c1 * random.uniform(0, 1) * (pbest[i] - X[i]) + c2 * random.uniform(0, 1) * (gbest - X[i])  ww = 1  for k in range(dim):  if DOWN[k] < X[i][k] + V[i][k] < UP[k]:  continue  else:  ww = 0  X[i] = X[i] + V[i]*ww  fitness.append(fit)  print('Running time: ', time.time() - t1) |
